# Possible neural mechanisms of psychotherapy for trauma-related symptoms: cerebral responses to the neuropsychological treatment of post-traumatic stress disorder model individuals

**DOI:** 10.1038/srep34610

**Published:** 2016-10-04

**Authors:** Tamaki Amano, Motomi Toichi

**Affiliations:** 1 Graduate School of Medicine, Kyoto University, Kyoto, Japan; 2 The Organization for Promoting Neurodevelopmental Disorder Research, Kyoto, Japan.

## Abstract

Psychotherapy is often effective for treating psychogenic disorders, but the changes that occur in the brain during such treatments remain unknown. To investigate this, we monitored cerebral activity throughout an entire session using a psychotherapeutic technique in healthy subjects. Since post-traumatic stress disorder (PTSD) is a typical psychogenic psychiatric disorder, we used PTSD-model volunteers who had experienced a moderately traumatic event. The technique used as psychotherapy was eye movement desensitisation and reprocessing (EMDR), a standard method for treating PTSD. The oxygenated haemoglobin concentration ([oxy-Hb]), a sensitive index of brain activation, measured using multi-channel near-infrared spectroscopy, revealed changes in [oxy-Hb] in the superior temporal sulcus (STS) and orbitofrontal cortex (OFC). During a vital therapeutic stage, a significant reduction in the activation by forced eye movements was observed in the right STS, and a trend toward a reduction in the left OFC. The hyperactivation of the right STS on the recall of unpleasant memories, and its normalisation by eye movements, seem to reflect an important neural mechanism of the psychotherapy. These findings suggest that psychotherapy for traumatic symptoms involves brain regions related to memory representation and emotion, and possibly those that link memory and emotion, such as the amygdala.

A variety of studies conducted over a substantial period of time have found the effects of psychotherapies to be generally positive^1^. Although research on the neuroscientific correlates of psychotherapies has yielded enormous progress, we still cannot provide an evidence-based explanation of how or why psychotherapeutic interventions lead to change, namely, the mechanisms through which treatments operate. A thorough investigation of the neural effects of psychotherapy is needed to provide a neurobiological foundation for the treatment protocols used in psychotherapy. However, because psychotherapies differ in terms of how they approach or treat each disorder, the neural mechanisms of treatment naturally differ^2^. Thus, despite recent developments in neuroimaging, it has been very difficult to determine the neural mechanisms of psychotherapy.

Trauma-related disorders, such as post-traumatic stress disorder (PTSD), have seemed particularly suitable for research on the neural mechanisms of psychotherapeutic treatment. This is because PTSD is one of the typical psychogenic psychiatric disorders; its symptoms, such as flashbacks, arousal and nightmares, are obvious, and the cause of the disorder can generally be determined^3^. Furthermore, a considerable number of neuroimaging results from PTSD studies have been published. Several studies have localised the dysfunction in PTSD patients to the limbic, paralimbic, and prefrontal structures: the deactivation of the anterior cingulate and frontal regions and the activation of the temporal cortex and insula^4^,^5^,^6^,^7^,^8^. Changes in the activity in these regions seem to be related to difficulties in attention, arousal regulation, emotional/self-awareness, or social and emotional processing^9^. Thus, PTSD and related conditions seem to be good targets for neuropsychological research, as they are characterised by a relatively localised neural background. However, the mechanisms by which psychotherapies are often effective in the treatment of PTSD and the nature of the changes occurring in the brain during such treatment remain unknown.

There are some difficulties in conducting research on the neural mechanisms of psychotherapy. For example, most clinical studies on therapy are often difficult to conduct under an experimental condition in which brain activities are measured. Another problem is the period needed for the psychological treatment. Indeed, such treatment usually takes more than days, and it sometimes involves ambiguous psychological changes. Additionally, because the content of psychological interventions often differ according to the patient, it is difficult to control the paradigm that is being used. The protocols of psychotherapies (particularly psychodynamic therapies) are often unstructured, rendering their content complex. In contrast, EMDR and cognitive behavioral therapy (CBT) are typically significantly more structured than psychodynamic therapies, and follow strict protocols.

To avoid these methodological problems, we focused on eye movement desensitisation and reprocessing (EMDR)^10^,^11^, one of the standard therapeutic methods for treating trauma-related symptoms recommended in a guideline of the American Psychological Association (APA)^12^, which involves a clearly structured protocol consisting of eight phases^10^,^11^,^13^. EMDR extinguishes symptoms very rapidly, often in only one session^14^. Therefore, EMDR is considered to be suitable for investigating the changes in the brain related to psychological interventions. Several neuroimaging studies of EMDR have investigated the effects of this therapy on patients with PTSD; they found changes in the activation of the brain after (not during) treatment sessions using single-photon emission computed tomography (SPECT), magnetic resonance imaging (MRI), and positron emission tomography (PET)^15^,^16^.

A further methodological problem concerns techniques to monitor cerebral activities. These measurements should be performed by a therapist in an interview setting, and the cerebral activities of the subject should be monitored throughout the therapy session. We used near-infrared spectroscopy (NIRS), which is a non-invasive optical technique for monitoring cerebral haemodynamic changes. In our previous multi-channel NIRS study^17^,^18^, we examined a patient who suffered from PTSD following a surgical accident^17^. During the EMDR session, haemoglobin levels in the areas from the frontal to the temporal lobes were measured using NIRS. The oxygenated haemoglobin ([oxy-Hb]) level is a sensitive index of brain activation, and it was found to increase markedly in the right superior temporal sulcus (STS; Brodmann’s area (BA) 21) and the lateral orbitofrontal cortex (OFC; BA 10) during recall of a traumatic episode. Thus, it appears to be important to examine the changes in blood flow during a treatment session to elucidate the neurobiological mechanisms of psychological treatments. Although our previous case studies indicated that the STS and OFC are involved, other previous NIRS studies have argued that the important region is the DLPFC^19^,^20^,^21^. For these reasons, we focused on the STS, the OFC and the DLPFC.

In this study, we employed “PTSD-model” subjects who were basically healthy with unpleasant autobiographical memories (traumatic life events)^22^. It was expected that they would be free from the confounding symptoms (e.g., depression, anxiety, and tension) sometimes seen in patients with PTSD.

## Results

### Clinical effects of EMDR

All seven subjects were able to complete the planned protocol. Table 1 presents data regarding the subjects and their Subjective Units of Disturbance Scale (SUDS) and Validity of Cognition (VOC) scale scores; two scales for evaluation of the therapeutic effect. The mean SUDS score after the session (1.1) was significantly lower (t = 10.824, p < 0.01) than that before the session (8.0). The mean VOC score after the session (6.1) was significantly higher (t = 22.000, p < 0.01) than that before the session (3.0). Although the original EMDR protocol for a PTSD patient with clinical symptoms requires a reduction in the SUDS score to 0 (or 1 if 0 is not ecologically valid) and an increase in the VOC score to 7, the changes in the SUDS and VOC scores of our subjects were large enough for the therapeutic session to be regarded as successful given the low level of severity of their traumatic experiences and our use of only one brief session. The mean Profile of Mood States (POMS) scores (with the exception of that for “vigour-activity”) (n=5) were lower after the session than before the session (tension-anxiety: 7.2 and 3.4, depression-dejection: 2.6 and 0.6, anger-hostility: 1.2 and 0, fatigue-inertia: 3.2 and 1.4, and confusion-bewilderment: 4.6 and 2.4, respectively). The “vigour-activity” score was higher after the session (9.6) than before (8.4). The results of the paired t-tests reflected a strong tendency (p < 0.1) toward a significant difference in the depression-dejection (t = 2.449, p = 0.070) and confusion-bewilderment (t = 2.750, p = 0.051) scores. These changes indicated that the EMDR session resulted in a better subjective mood, reflecting a reduction in the perceived unpleasantness of the events recalled and an increase in positive feelings. These POMS results also suggest the success of the EMDR session.

### Heart rate data

**Figure 1:**
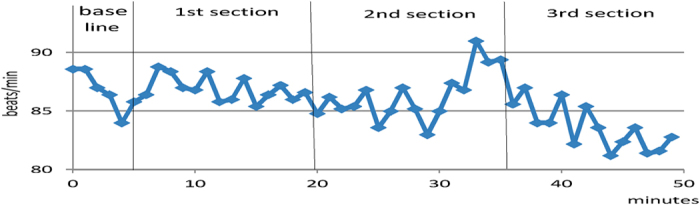
Changes in heart rate during the session (n = 6; data on one subject were not available). Visual global inspection reveals a gradual decrease in heart rate through the whole session, which suggests a normalization of autonomic hyperarousal. Detailed inspection shows a temporal increase in heart rate upon recall of unpleasant memories. During the second section which focused on the unpleasant memories, heart rate reached the highest level, and then it rapidly decreased to a normal level in the third section. Abscissa: time (minutes). Ordinate: heart rate (beats per minute).

When the second section focused on the unpleasant memory and emotions, the subjects’ heart rate increased gradually as they recalled the memories (Fig. 1). During the third section, installation, their heart rate decreased gradually to a normal level. These changes in heart rate suggest that the autonomic states of the subjects were elevated during recall but stabilised after desensitisation, which is consistent with the change in emotional states assessed by the SUDS.

### NIRS data

Visual inspection revealed that two of the three cerebral regions on which we focused showed changes in [oxy-Hb] levels: the right STS and the bilateral OFC. The grand-average [oxy-Hb] level under the Recall condition increased, and it was maintained at a high level and then decreased under the EM condition. In a pre-study (in six healthy subjects who were different from those in the main study), it was confirmed that no significant changes due to EM were found.

**Figure 2:**
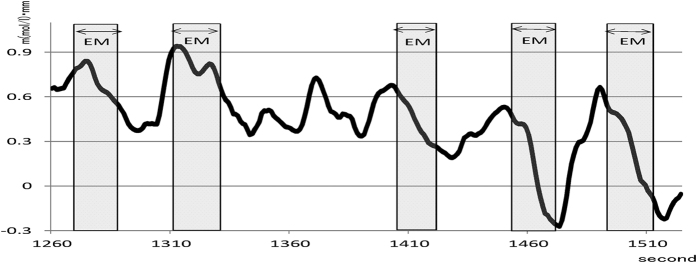
An example of the change in [oxy-Hb] during a session of desensitization using eye movements. Graph shows an example of the change in [oxy-Hb] levels from 20th to 26th minute in channel 43 (superior temporal sulcus, STS), in one subject (No. 6). The horizontal axis shows the passage of time (seconds) from the start. The light shading in the graph shows the period of eye movements (EMs), usually 20–30 seconds. The [oxy-Hb] level increased rapidly upon recall of an unpleasant memory, and then it suddenly decreased as soon as the EM started. As this process was repeated, the baseline [oxy-Hb] level gradually decreased. The other subjects also demonstrated a similar pattern of [oxy-Hb] changes. Abscissa: time (second). Ordinate: haemoglobin concentration (m(mol/l)•mm).

Figure 2 shows an example of the change in the [oxy-Hb] levels in Ch 43 (right STS) in one subject (No. 6). The graph clearly shows the relationship between the changes in [oxy-Hb] levels and EM. The [oxy-Hb] level increased rapidly upon recall of an unpleasant memory, but it suddenly decreased substantially as soon as the EM started. As this process was repeated, the baseline [oxy-Hb] level gradually decreased. The other subjects also demonstrated a similar pattern of [oxy-Hb] changes.

**Figure 3:**
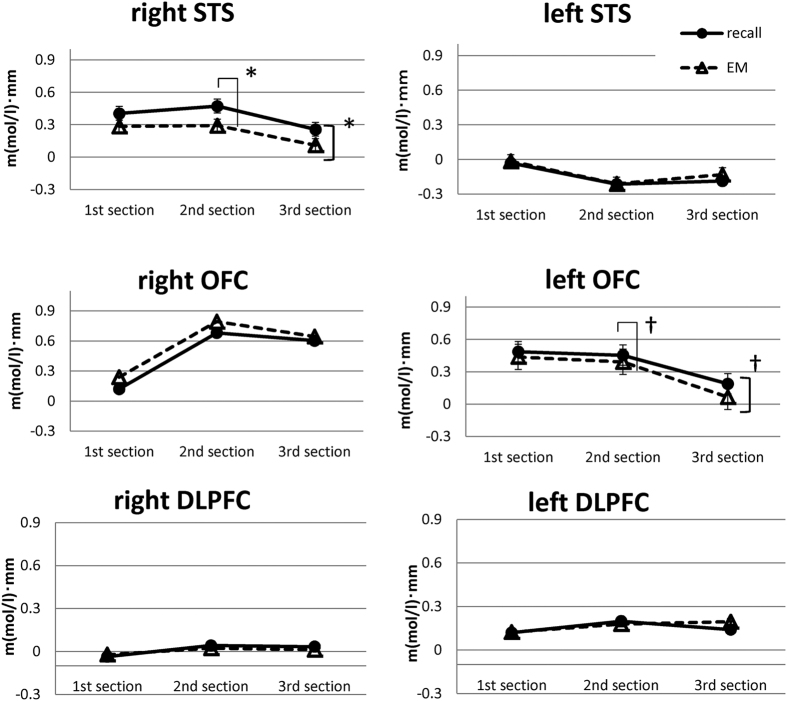
Mean [oxy-Hb] levels under Recall and EM conditions in the superior temporal sulcus (STS), the orbitofrontal cortex (OFC), and the dorsolateral prefrontal cortex (DLPFC). Mean change in [oxy-Hb] levels under each condition ([Recall condition], [EM condition]) in the left and right STS, the OFC and the DLPFC are shown. Repeated ANOVA revealed significant effects of the conditions in the right STS [F (1, 6) = 6.482, p = 0.044, ηp2 = 0.519] (p < 0.05) and the left OFC [F (1, 6) = 4.407: p = 0.081, ηp2 = 0.423] (p < 0.1). The post hoc test for multiple comparisons revealed significant differences during the second section in the right STS (p = 0.024, p < 0.05) and the left OFC (p = 0.084, p < 0.1). No significant differences were observed in the left STS, the right OFC, the left DLPFC or the right DLPFC. Ordinate: haemoglobin concentration (m(mol/l)•mm).

We divided the [oxy-Hb] data from the regions of interest according to section (first section: assessment, second section: desensitisation, third section: installation) and condition (Recall, EM). Figure 3 shows the changes in [oxy-Hb] levels in the bilateral STS, bilateral OFC, and bilateral dorsolateral prefrontal cortex (DLPFC). A two-factor repeated-measures analysis of variance (ANOVA) (section × condition) revealed a significant main effect of condition ([Recall condition] > [EM condition]) in the right STS [F (1, 6) = 6.482, p = 0.044, ηp2 = 0.519], and the post hoc tests showed a significant difference during the second section (p = 0.024). This indicates that, as subjects recalled unpleasant memories (images), their [oxy-Hb] level in the right STS initially increased and then immediately decreased with EM. The reduction with EMs in the right STS was significant in the second section (in the desensitisation phase). A strong tendency toward a difference according to condition ([Recall condition] > [EM condition]) was observed in the left OFC [F (1, 6) = 4.407: p = 0.081, ηp2 = 0.423], and post hoc tests showed a tendency toward a difference during the second section (p = 0.084). Regarding the other brain regions, no significant main effect of section and no interactions between condition and section were observed.

## Discussion

The psychological data collected and changes in heart rate observed in this study suggested that treatment with EMDR was successful. Because the EMDR session was successful in all seven PTSD-model subjects, the observed cerebral activation was considered to reflect the therapeutic process of EMDR. It is possible that our data reflect only the neuropsychological mechanism of EMDR, because the subjects were free from the possible confounding factors found in previous studies with PTSD patients, such as complications or symptoms secondary to PTSD (e.g., depression, anxiety, and tension). Several previous neuroimaging studies using SPECT, MRI, and PET to investigate the psychological effects of EMDR have shown changes in the activation of the brain after treatment but no research has investigated changes associated with ongoing sessions. For this reason, it has been difficult to use these neuroimaging modalities for the direct monitoring of the changes in blood flow that reflect the therapeutic process itself. Therefore, one of the imaging techniques suitable for revealing the therapeutic mechanisms in the brain is continuous haemodynamic monitoring using multi-channel NIRS.

There were two major findings related to overall regional changes in blood flow.

First, the area around the right STS and the bilateral OFC showed higher activation than other regions during EMDR. The [oxy-Hb] levels in the six regions of interest (bilateral STS, OFC and DLPFC) changed as the session progressed (see Fig. 3). In most regions, these levels were high before desensitisation and then decreased gradually during desensitisation, installation, and the elicitation of a positive cognition. With the exception of the left STS, the [oxy-Hb] changes seemed to be generally synchronised with the autonomic arousal level, as indicated by heart rate. Because the changes in heart rate seemed to reflect the autonomic states of the subjects, these [oxy-Hb] changes seem to be synchronised with emotion^23^.

Second, there was a significant difference between the activation in the right STS during desensitisation under the Recall versus under the EM condition. In short, the reduction with EMs in the right STS was significant in the second section (the desensitisation phase). In the left OFC, there was also a trend toward a change similar to that observed in the right STS. Another important finding is the relationship between brain activation and EMs. The right STS was strongly activated upon recall of traumatic memories, with a sudden reduction with EMs. The role of EMs in this regard seems to involve normalising the hyper-activation of those brain areas that were stimulated by the recall of an unpleasant memory. This suggests that EMs may reduce hyper-activation of the right STS.

In what follows, we discuss the six regions of interest and the possible mechanisms of action of EMDR.

The first region is the STS. The right STS was more highly activated than the left STS during visual recall (see Fig. 3). A significant reduction in activity was found in the right STS during the EMDR desensitisation phase (the second section in Fig. 3). These results suggest that the right STS is an important region involved in EMDR. The roles of the STS in human cognitive functioning have been gradually elucidated. Recent studies have found a relationship between the STS and memory representation^24^, as first described in the classic work of Penfield^25^. Consistent with the present results, our previous case studies using NIRS^17^ with subjects who had memories of traumatic events (following a surgical accident) showed strong activation of the right STS during the recall of visual scenes. Significant reductions in the [oxy-Hb] levels in the right STS were correlated with reductions in the vividness of visual traumatic scenes during EMDR sessions^17^,^18^. Therefore, we believe that the strong activation in the right STS reflected the vivid recall of unpleasant memories and that the reduction in activation during EMs reflected a reduction in vividness^26^. Interestingly, no such change was observed in the left STS. Because both emotional processing and visual processing are generally lateralised to the right hemisphere^27^,^28^, it is possible that unpleasant visual memories were represented in the right rather than the left STS in our subjects.

The second region is the OFC. The average activation was higher in and around the bilateral OFC regions than in other regions, with no significant difference between the left and right OFC (see Fig. 3). The OFC is known to be important for emotional response and arousal^29^. Therefore, the overall increase in blood flow in both parts of the OFC is considered to reflect a negative emotional state attributable to unpleasant memories. The elevated autonomic arousal reflected in the heart rate data may also be related to activation of the OFC^30^.Converging evidence suggests that the central and lateral parts of the OFC are specifically involved in representing the emotional impact of anticipated negative outcomes. For example, a lesion study supported the involvement of the OFC in emotional processing^31^. Additionally, according to our previous studies^17^, the high level of [oxy-Hb] in and around the OFC area was correlated with the occurrence of an unpleasant emotion. Therefore, it is safe to assume that the right OFC reflects subjects’ negative emotional states. In other words, activation of the OFC may constitute an epiphenomenon of emotional state. Since emotional reactions vary greatly among individuals, it may be difficult to detect statistically significant differences in our small sample.

However, a trend toward a reduction in activation in the left OFC due to EMs was observed only in the second section (see Fig. 3). The co-occurrence of the reduction in the activation of the left OFC and the right STS may be related to the change in emotional states associated with an unpleasant memory. Several studies have reported functional differences between the left and right OFC, with the left OFC involved in the regulation of emotion and arousal^32^. In the second section, EMs reduced the vividness of (and negative emotion associated with) an unpleasant memory, which, in turn, would be expected to reduce the need to regulate the emotion, as reflected in the reduction in left OFC activation. Thus, a trend toward a reduction in cortical activation in the left OFC due to EMs may reflect a reduced need to regulate emotional responses due to desensitisation.

The third region is the DLPFC. The bilateral DLPFC showed no significant changes in [oxy-Hb] levels in this study (see Fig. 3). However, this result differs from those of previous NIRS studies measuring the prefrontal cortex (PFC). In these studies, subjects with PTSD showed significant activation in the DLPFC during exposure to trauma-related visual stimuli^19^,^20^; this activation then decreased after EMDR^21^. These issues regarding this apparent difference should be considered. First, there were differences between the subjects in the studies; our subjects were volunteers with moderately traumatic experiences but not PTSD. The second issue concerns differences in the design of the studies: whereas we measured a wide cortical area, including frontal and temporal regions, they measured only the PFC. For this reason, we were able to evaluate changes in regional blood flow from a more global perspective. The DLPFC generally has a role in generating behaviour that is flexible and adaptive in accordance with current sensory input^33^. Other studies on the mechanisms underpinning EMDR have claimed that the DLPFC plays an important role in the treatment of PTSD^21^; however, we observed no activation of the DLPFC during the session. Furthermore, when we applied on-the-spot EMDR to patients with severe dementia, all showed marked improvement in their PTSD-like syndrome^34^. If DLPFC responsiveness is an essential requirement for the effectiveness of EMDR, our patients with dementia who had severely impaired DLPFC functioning would not be expected to show improvement. Thus, the DLPFC seems to play a lesser role in the desensitisation phase of an EMDR session than would be expected from some previous studies.

In addition to the three regions studied (STS, OFC and DLPFC), other regions may be involved in the changes in brain functioning associated with EMDR therapy. One such region may be the amygdala (AMG), which is anatomically connected to the OFC and STS^35^,^36^. According to an animal model of classical fear conditioning, the AMG serves as the central intersection where information from all senses is connected and endowed with emotional meaning^37^,^38^,^39^,^40^. Since PTSD is a form of fear conditioning, the AMG is thought to play an important role in producing PTSD symptoms. An important study on fear conditioning reported that reconsolidation (i.e., re-encoding) while activating (recalling) a fearful memory prevents the return of fear^41^. This study suggested an important clinical implication for the treatment of PTSD because the AMG is involved in fear conditioning^42^.In other words, the eye movement technique is more than a habitual procedure; rather, it is an important technique for de-conditioning via activation of the amygdala.

Previous cognitive neuroscientific studies have shown that the AMG is also related to EM and attention^43^. For example, Okada *et al*.^44^. Showed that reflexive EM (joint attention) is mediated by the AMG, suggesting a close link between EM and the AMG. The AMG is also known to be related to the STS^45^,^46^. Recent studies have shown a functional link between the STS and the AMG, which would seem to be consistent with the present results^47^,^48^,^49^, provided evidence for a neurobiological basis of the bilateral alternating stimulation, suggesting that the increase in right AMG activation along with the decrease in left DLPFC activation may facilitate the therapeutic reintegration of information^49^. Other areas, such as limbic structures (e.g., the hippocampus and anterior cingulate cortex), may also play an important role in the pathophysiology of PTSD and its treatment^50^,^51^.

We discuss the possible therapeutic mechanisms of action. Based on the findings of this study and the above neuroscientific evidence, one possible mechanism underpinning the main therapeutic stage of EMDR (phase 4; the desensitisation phase) would be as follows: while traumatic memory is activated, induced EMs block the over-representation of the traumatic memory through a forced attentional shift, while they simultaneously ameliorate the conditioned fear by stimulating the AMG^49^. This would result in the reduced invasiveness of the traumatic memory. Alternatively, it is possible that the involvement of the OFC and DLPFC might be epiphenomenal, varying across patients or pathologies. In our previous study, we applied EMDR to patients with severe late-stage dementia. All patients showed marked improvement in symptoms of fear arousal, such as repeated outbursts of screaming^34^. For these patients, EMDR was apparently effective despite the remarkable cognitive impairment due to the pathological atrophy in the brain, particularly in the DLPFC and temporal cortex. The evidence of little involvement of the DLPFC in the core therapeutic process of EMDR seems consistent with the results of the present study.

The current findings appear to indicate that the main psychotherapeutic stage involves both cognitive and emotional operations (i.e., focusing on past experience (memory representation) and detaching the experience from negative emotion) in which forced attentional shifts induced by EMs play an important role.

This study has several limitations. First, we used NIRS, which does not measure the deep brain regions (e.g., limbic areas) but does measure an area from the prefrontal to the inferior temporal cortices. Other brain areas should be examined in future research. Second, our subjects were not patients diagnosed with PTSD. It would be interesting to examine whether the present findings could be replicated in a clinical population (i.e., patients with PTSD). Third, our sample was small, and the visual memories of the subjects were accompanied by strong traces of previous experiences. Future studies should examine a larger number of subjects with different types of memory. We need to examine more subjects to confirm the present findings and clarify the relevant mechanisms of action.

## Methods

### Ethics statement

This study was approved by the ethics committee of the Kyoto University Graduate School of Medicine and was conducted in accordance with the principles of the 1964 Declaration of Helsinki. All subjects were naive to the experiment and research setting and provided written informed consent before the start of the experiment.

### Subjects

The inclusion criteria were: 1) aged at least 20 years and less than 60 years; 2) being strongly right-handed. The exclusion criteria were: 1) being a smoker; 2) a history of drug or alcohol abuse; 3) taking any medication; 4) a history of psychiatric illness (including schizophrenia, bipolar disorder, depression and obsessive-compulsive disorder) or neurological disorders (such as parkinsonism and cerebrovascular diseases. The absence of psychiatric or neurological symptoms was confirmed via interviews with an expert psychiatrist (i.e., M.T., one of the authors).

Seven subjects (four women, three men; mean age 34.4 (range 22–55) years) who were able to recall “a memory that feels unpleasant now” were recruited. We used volunteers without psychiatric disorders, such as depression or anxiety disorders, to focus on the effect of EMDR on moderate traumatic memories. The primary reason why we recruited PTSD-model subjects rather than patients with PTSD was that our experimental paradigm required that the main therapeutic stage (phase 4 below) involving EMs be completed in one brief session. It is not unusual for phase 4 to be repeated several times over a period of weeks or months when patients with PTSD are treated using this modality. Furthermore, patients with PTSD often suffer from other psychiatric or physical complications (e.g., depression, social anxiety, dissociation, panic attacks, and autonomic instability) that may compromise data regarding brain activity during the main therapeutic period. We assumed that the participants’ brain activity during EMDR reflected processes occurring during the therapeutic process because all of the subjects were free from confounding psychiatric pathology. All were right-handed. Other demographic data are presented in Table 1.

### EMDR session

A standard EMDR protocol includes eight phases^10^,^11^,^13^. Phase 1 involves gathering data about the patient’s history and clinical symptoms. Phase 2 involves establishing a therapeutic framework and defining the appropriate level of expectations. Phase 3 focuses on assessing trauma-related symptoms that involve vivid visual imagery associated with traumatic memories, a positive and a negative belief about the self, relevant emotions, and bodily sensations (assessment phase). Phase 4 is the primary therapeutic stage, in which alternate bilateral stimulation (EM etc.), usually lasting 20–30 s, is used to ameliorate traumatic reactions (desensitisation phase). Phase 5 involves cognitive modification, in which positive cognition (i.e., a “sense of safety” or a “feeling of being loved”) is instilled (installation phase). Phase 6 involves a scan of bodily sensations to identify pain or abnormal experiences. Phase 7 is the closure phase, and Phase 8 is used to prepare for the next session.

**Figure 4:**
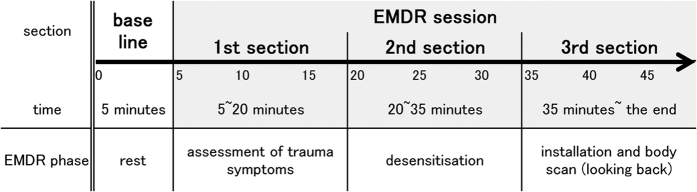
The schema of the study procedure. The NIRS measurement and its relation to the EMDR sessions are shown. Thick line indicates the monitoring using NIRS.

In this study, only one session, involving phases 3 (assessment phase), 4 (desensitisation phase), 5 (installation phase), and 6 (body scan phase), was conducted. The procedure is shown in Fig. 4.

### Measurements

#### Evaluation of the therapeutic effect (SUDS, VOC)

Two subjective scales were used to examine the effect of one session. The SUDS assesses the intensity of negative affect on a 10-point scale on which 0 indicates no discomfort and 10 indicates the highest level of discomfort or distress imaginable. The VOC scale, which measures the believability of a suggested positive cognition, uses a seven-point scale on which 1 indicates that the cognition feels completely false and 7 indicates that it feels completely true to assess self-recognition regarding the positive cognition (e.g., safety, self-affirmation). Positive cognitions improve an individual’s psychophysiological state and enhance their ability to engage in more adaptive self-soothing personal and social behaviours in the face of various PTSD “triggers”. Such cognitions act as the mirror image of, or an antidote to, negative (distorted) cognitions. The SUDS and VOC scores reflect how the person ‘feels’ not ‘thinks’. During the assessment phase (phase 3), two baseline scores are obtained. If the EMDR session succeeds, the SUDS score will decrease to 0 and the VOC score will increase to 7. POMS is a standard validated psychological test, originally formulated by McNair *et al*.^52^. The questionnaire contains 65 items measuring six mood states: tension, depression, anger, vigour, fatigue and confusion. Items are rated on a 5-point Likert scale ranging from 0 (not at all) to 4 (extremely). POMS is a measure of how subjects feel before and after a session.

The means of the SUDS, VOC, and POMS scores before and after sessions were compared using paired *t*-tests. Average differences between the SUDS and VOC scores with *p*-values <0.05 were considered to be statistically significant, and those with p-values <0.1 were considered to show a strong tendency toward a significant difference. The results of the SUDS and VOC are presented in Table 1.

#### Heart rate

To monitor autonomic arousal, heart rate was measured throughout the session using a light sensor attached to the index finger. Heart rate data were measured in six subjects (n = 6). All sessions were recorded using a video camera.

#### NIRS measurement

**Figure 5:**
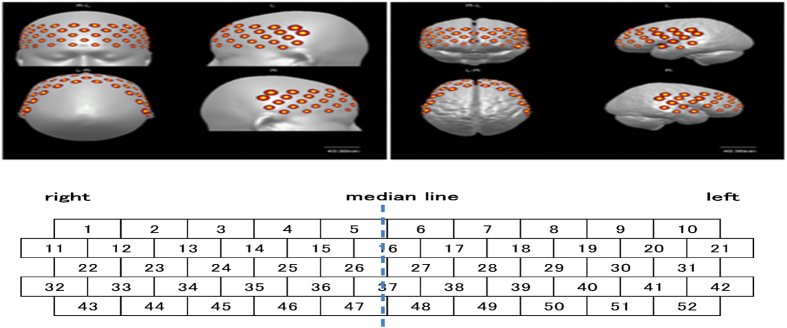
The position of the NIRS probe and the area measured (Tsuzuki *et al.*^58^).

The procedure is shown in Fig. 4. Cerebral activation during the EMDR session was monitored using NIRS, an optical neuroimaging technology for the non-invasive detection of regional changes in blood flow^53^,^54^,^55^,^56^. We used a 52-channel NIRS instrument (ETG-4000; Hitachi Medical, Tokyo, Japan), and the distance between the emitter and detector was 30 mm. Combinations of the 52 nearest-neighbour pairs of input and output fibres were used to obtain topographical images of the area from the frontal lobe to the temporal lobe. The measurement point was the central point between the emitter and detector. Figure 5 shows the measurement points. The centre of the lowest row of the 3 × 11 holder was placed on the middle of the forehead (Fpz) according to the International 10–20 system for the electroencephalogram (EEG)^57^. The probes in the lowest row were placed horizontally along the reference curve (i.e., the line that links Fp1 to Fp2). The bottom edge of the probes was located on the top edge of the eyebrow^58^.

Regional cerebral blood flow was measured in terms of changes in [oxy-Hb] and deoxygenated haemoglobin ([deoxy-Hb]) levels. In this study, changes in [oxy-Hb] levels, the most sensitive indicator of changes in regional cerebral blood flow, were used as the index of activation^59^. The baseline [oxy-Hb] level was the average value during the last 5 s of the 5-min rest period after placing the probes on the head. Changes in the [oxy-Hb] level were measured every 0.1 s.

### Data processing and analysis

The correspondence between the NIRS data and the temporal course of the sessions was checked carefully using video records. The EMDR session was divided into three sections: the first section (minutes 5 to 20) involved the assessment of trauma symptoms (phase 3); the second section (minutes 20 to 35) involved desensitisation (phase 4); and the third section (minute 35 until the end) involved installation (phase 5) and a body scan (phase 6) (Fig. 4). Owing to the nature of the study, it was unreasonable to expect the responses of the subjects during each phase to be uniform. However, we were able to recognise changes in blood flow that corresponded to each therapeutic process.

Data were collected under two conditions: the first condition [Recall condition] involved recalling unpleasant memories (without EM), and the second condition [EM condition] involved recalling unpleasant memories with EMs induced by the experimenter (an EMDR therapist). The Recall condition was defined as the 15 seconds preceding the start of the EMs, and the EM condition was defined as the 15 seconds following the start of the EMs.

Bergmann^15^, who conducted a comprehensive review of neurobiological studies on EMDR, and Boccia^60^ identified the main regions of changes after treatment with EMDR. Based on these studies, as well as on our previous studies^17^,^18^, we focused on the following regions of interest in this study: the left and right DLPFC (Ch 29 and Ch 24, respectively), OFC (Ch 48 and Ch 47, respectively), and STS (Ch 52 and Ch 43, respectively). Ch 29/24, Ch 48/47, and Ch 52/43 correspond to BA 45/46, BA 10/11, and BA 21, respectively. Two-factor repeated-measures ANOVA was used to examine differences in the changes in [oxy-Hb] levels among sections (first, second, and third sections) and between conditions (Recall and EM conditions) in each brain region. *Post hoc* multiple comparisons were performed using the Bonferroni method (p < 0.1). Differences at the level of p < 0.05 were considered statistically significant, and those at the level of p < 0.1 were considered to indicate a trend toward a difference. We used SPSS ver. 22 (IBM; Armonk, NY, USA) for the analyses.

### Pre-study

The pre-study for the effect of EM was conducted under a no-recall condition in order to examine whether there would be an artefact due to EM or EM itself induce any cerebral activation. Six subjects (one woman, five men; mean age 33.1 (range 20–50) years) were examined under two conditions: an eye fixation condition and an EM condition; each subject completed three trials under each condition.

## Additional Information

**How to cite this article**: Amano, T. and Toichi, M. Possible neural mechanisms of psychotherapy for trauma-related symptoms: cerebral responses to the neuropsychological treatment of post-traumatic stress disorder model individuals. *Sci. Rep.* **6**, 34610; doi: 10.1038/srep34610 (2016).

## Figures and Tables

**Table 1:** Characteristics of Subjects.

No.	Age	Gender	Occupation	Theme of session	SUDs (0–10)			VOC (1–7)		
					Before	After	Before	After	
1	30	male	Caregiver	Death of father	8	2	3	6		
2	35	male	Care manager	Death of mother	8	2	3	6		
3	55	female	Office worker	Death of sister	10	2	3	6		
4	22	female	Caregiver	Unfaithful partner	6	2	3	6		
5	27	male	Office worker	Broken marital engagement	9	0	3	6		
6	22	female	College student	Groping incident	8	0	3	7		
7	50	female	Caregiver	Son’s accident	7	0	3	6		
Ave.	34.4				8.0	1.1^a^	3.0	6.1^a^		
S.D.	12.2				1.2	1.0	0.0	0.3		

^a^p < 0.01.

SUDS: Subjective Units of Disturbance Scale.

VOC: Validity of Cognition.
